# Acute Hypoglycemia Induces Retinal Cell Death in Mouse

**DOI:** 10.1371/journal.pone.0021586

**Published:** 2011-06-27

**Authors:** Martine Emery, Daniel F. Schorderet, Raphaël Roduit

**Affiliations:** 1 Institute for Research in Ophthalmology (IRO), Sion, Switzerland; 2 Department of Ophthalmology, University of Lausanne, Lausanne, Switzerland; 3 Ecole Polytechnique Fédérale de Lausanne (EPFL), Lausanne, Switzerland; Newcastle University, United Kingdom

## Abstract

**Background:**

Glucose is the most important metabolic substrate of the retina and maintenance of normoglycemia is an essential challenge for diabetic patients. Glycemic excursions could lead to cardiovascular disease, nephropathy, neuropathy and retinopathy. A vast body of literature exists on hyperglycemia namely in the field of diabetic retinopathy, but very little is known about the deleterious effect of hypoglycemia. Therefore, we decided to study the role of acute hypoglycemia in mouse retina.

**Methodology/Principal Findings:**

To test effects of hypoglycemia, we performed a 5-hour hyperinsulinemic/hypoglycemic clamp; to exclude an effect of insulin, we made a hyperinsulinemic/euglycemic clamp as control. We then isolated retinas from each group at different time-points after the clamp to analyze cells apoptosis and genes regulation. In parallel, we used 661W photoreceptor cells to confirm *in vivo* results. We showed herein that hypoglycemia induced retinal cell death in mouse via caspase 3 activation. We then tested the mRNA expression of glutathione transferase omega 1 (Gsto1) and glutathione peroxidase 3 (Gpx3), two genes involved in glutathione (GSH) homeostasis. The expression of both genes was up-regulated by low glucose, leading to a decrease of reduced glutathione (GSH). *In vitro* experiments confirmed the low-glucose induction of 661W cell death via superoxide production and activation of caspase 3, which was concomitant with a decrease of GSH content. Moreover, decrease of GSH content by inhibition with buthionine sulphoximine (BSO) at high glucose induced apoptosis, while complementation with extracellular glutathione ethyl ester (GSHee) at low glucose restored GSH level and reduced apoptosis.

**Conclusions/Significance:**

We showed, for the first time, that acute insulin-induced hypoglycemia leads to caspase 3-dependant retinal cell death with a predominant role of GSH content.

## Introduction

Neural tissue, including retina, is totally dependent on glucose for normal metabolic activity. Since the level of glucose storage is negligible compared with the eye glucose demand, this tissue is dependent on glucose delivery by circulating blood. In both type I and II diabetes, normalization of blood glucose concentration is an important issue to avoid secondary long-term microvascular complications, including nephropathy, cardiovascular disease, neuropathy and retinopathy [1]. Although diabetes-related eye diseases are generally linked to hyperglycemia [2], iatrogenic hypoglycemia causes morbidity in most people with type I diabetes and in many with advanced type II diabetes [3]. Diabetic retinopathy is the results of microvascular retinal changes promoted by hyperglycemia through the formation of advanced glycation end products, resulting in weakening and blockage of blood vessels through up-regulation and secretion of vascular endothelium growth factor (VEGF) [1], [4]. The role of hyperglycemia in the retina via pericyte apoptosis and in vascular complications has been extensively studied in a large number of *in vivo* and/or *ex vivo* models [5], [6].

While hyperglycemia is an accepted and well-investigated cause of diabetes-related eye diseases, few studies exist which implicate hypoglycemia as a key factor involved in visual disorders. The majority of data has focused on *in vitro* or *ex vivo* studies: Luo *et al.* showed that conditions of low glucose reduced viability of all retinal cell types in a mixed primary cell culture [7] and Zeevalk and Nicklas demonstrated the sensitivity of isolated chick retinas to *in vitro* aglycemic conditions [8]. More recently, Umino *et al.* showed that chronic moderate hypoglycemia in mouse led to loss of vision and eventual retinal degeneration [9], while Punzo *et al.* suggested that cone death in retinitis pigmentosa could be, at least in part, the result of the starvation of cones via the insulin/mTOR pathway [10].

Glutathione (γ-L-glutamyl-L-cystein-glycine; GSH) is the most abundant non-protein thiol in the cell. It is involved in many cellular functions including regulation of DNA and protein synthesis, signal transduction, cell cycle regulation, as well as maintaining a stable thiol redox state by acting as an antioxidant and scavenger [11]. They are few cellular mechanisms that control intracellular levels of GSH (the reduced glutathione). Depletion of GSH occurs essentially in a reaction in which glutathione peroxidase (*Gpx*; E.C.1.11.1.9) reduces H_2_O_2_ by producing GSSG (the oxidized glutathione) and H_2_O, while NADPH is used to reduce back GSSG to GSH with the help of glutathione reductase (*GR*; E.C.1.8.1.9). Glutathione transferase (*Gst*; E.C.2.5.1.18) serves as a protective enzyme by adding GSH to proteins, targeting them for export from the cell. “Lost” GSH is replaced by direct uptake of GSH or by “*de novo*” synthesis through a two-step reaction utilizing L-glutamate, L-cysteine, glycine, ATP and both glutamate-cysteine ligase (*GCL*; E.C.6.3.2.2) and glutathione synthase (*GS*, E.C.6.3.2.3) (for review see [11], [12]).

Cell death, e.g. apoptosis and necrosis, is in constant balance with survival processes. Cells developed these signaling pathways in response to extra- or intracellular stresses, such as UV irradiation, starvation, growth factor deprivation, ER stress or pathogen infection. First, affected cells react by up-regulating genes implicated in cellular energy homeostasis and by adapting to the environmental stress. Then, cell death occurs through apoptosis or necrosis, two mechanisms leading to an irrevocable killing. The two main pathways of apoptosis are extrinsic and intrinsic as well as a perforin/granzyme pathway which, in turn, will activate caspase-3 [13]. In addition, caspase-independent mechanisms of neuronal cell death have also been identified.

We showed herein for the first time that acute insulin-induced hypoglycemia led to retinal cell death via an activation of caspase 3 pathway and a decrease of GSH content. We confirmed these results in photoreceptor 661W cells cultured at low glucose condition. In addition, replenishment of GSH, by addition of external glutathione ethyl ester protects the cells from low glucose-induced apoptosis, while a decrease of GSH content by inhibition of glutathione synthase at high glucose induced apoptosis. This study suggests that a strict control of the glycemia is critical for diabetic patients not only for brain defect but also for the maintenance of a good retinal function and vision and that glycemic excursion may not be harmless.

## Methods

### Mouse line

This study adhered to the Association for Research in Vision and Ophthalmology (ARVO) statement for the use of animals in ophthalmic and vision research and was approved by the Veterinary service of the State of Valais, Switzerland (permit ID: VS22). Wild-type 2-month-old C57BL/6 female mice (wt) were purchased from Charles River Laboratories (Les Oncins, France). Animals were kept in a 12-h light/12-h dark cycle with unlimited access to food and water.

### Hyperinsulinemic clamps

An indwelling catheter (Becton Dickinson AG, Basel, Switzerland) was inserted in the femoral vein of isoflurane-anesthetized mice, which were allowed to recover for 4 to 7 days. After a 5-h fasting period, awake and freely moving mice were subjected to 5 h of either a hyperinsulinemic/hypoglycemic or hyperinsulinemic/euglycemic clamp as described [14]. Mice were killed 4, 12 and 48 h post-clamp and isolated retinas were used to prepare retinal flat mounts, mRNAs and proteins for analysis.

### Cell Culture Conditions

Retinal explants were isolated from 2-month-old mice and cultured, on a nitrocellulose filter (Millicell, Millipore AG, Switzerland), in Dulbecco's modified Eagle's medium supplemented with 10% heat-inactivated fetal calf serum (both from Sigma) and 1% penicillin/streptomycin, for one-day prior to the experiment. Then, retinal explants were cultured in glucose-free DMEM supplemented with various glucose concentrations to achieve low (2 mM) or high (25 mM) glucose. Human retinal pigment epithelial cell line ARPE19 [15], 661W photoreceptor cell line [16] and human adult Müller glial cell line Mio-M1 [17] were cultured as described in their respective publications except for experiments at low and high glucose where cells were culture in glucose-free DMEM supplemented with various glucose concentration. One day before the experiment cells were synchronized with 2% FCS in complete high glucose (25 mM) medium for 24 h. Then, cells were cultured as indicated with various concentrations of glucose during diverse periods of time prior to analysis. Caspase 3 inhibitor Z-VAD-FMK was added at 10 µM for 48 h at 2 mM glucose before testing Caspase 3 activity and apoptosis by TUNEL assay. To modulate GSH level we cultured 661W photoreceptor cells at low (2 mM) or high (25 mM) glucose in absence or in presence of 200 µM buthionine sulphoximine (BSO) or 1 mM glutathione ethyl ester (GSHee) for 24 hours, then we analyzed for GSH level and cell apoptosis.

### Terminal dUTP Nick End-Labeling (TUNEL) of fragmented DNA

*In situ* cell death detection was performed 24 or 48 h after low glucose exposure, by TUNEL technology as described by the manufacturer (Roche Applied Science, Rotkreus, Switzerland) and detailed in Hamann S. *et al.* [18]. For each condition, apoptotic cells were visualized under a fluorescence microscope (Olympus BX51) using appropriate filters. Similar protocol was used on mouse flat-mounted retinas isolated 48 h after the clamp. Colorimetric TUNEL assay (Promega, Madison, WI, USA) was used on ten µm-embedded frozen sections of enucleated eyes, isolated from treated and control animals.

### FACS analysis

Relative cell death and apoptosis were assessed by staining with AnnexinV-FITC (IQ Products, Groningen, The Netherlands) and 7-AAD (Biolegend, San Diego, CA, USA) following the manufacturer's protocol. Samples were loaded on a FACScan (Becton Dickinson, San Jose, CA, USA) and analyzed with CellQuest (Becton Dickinson) or FlowJo (Treestar) software.

### Immunostaining

Enucleated eyes were fixed in 4% PFA/PBS for 45 min, followed by cryoprotection in 30% sucrose/PBS. Ten µm-embedded frozen sections were further processed for immunohistochemistry. Briefly, frozen retina sections were blocked in PBS with 3% normal goat serum (Sigma, Buchs, Switzerland) and 0.2% Triton X-100 (Sigma) for 1 h at RT and incubated with an antibody against cleaved Caspase 3 (dilution 1∶500, Cell Signaling Technology, Inc. Danvers, MA, USA) in the blocking buffer overnight at 4°C. Sections were incubated again in blocking buffer for 30 min at RT before being incubated with FITC Alexa-Fluor 594 goat anti-rabbit antibody (dilution 1/2′000) for 1 h at RT. Incubation with secondary antibody alone was used as a negative control. Tissue sections were counterstained with DAPI to identify retinal cell layers. 661W cells were cultured as described above and fixed in paraformaldehyde 4% during 45 min prior to be permeabilized and stained as described above.

### Western Blot Analysis

Thirty micrograms of proteins were electrically transferred to PVDF filters and incubated with anti–HIF3α (Rockland Immunochemicals Inc., PA, USA) and anti–tubulin (Sigma; St-Louis, MO). Secondary antibody, anti-rabbit-HRP (Amersham Biosciences; Otelfingen, Switzerland), was used to detect protein expression. Immune complexes were detected by chemiluminescence using LumiGLO (Amersham Biosciences, Otelfingen, Switzerland).

### Caspase 3 assay and superoxide detection

Caspase 3 activity was measured with a luminescent Caspase-Glo 3/7 kit (Promega, Madison, WI, USA) as described by the manufacturer. For superoxide detection, we used the MitoSOX™ detection reagent (Invitrogen, Basel, Switzerland) following the protocol described by the manufacturer. Briefly, we added 2.5 µM of the dye to the cells after exposition to low (2 mM) or elevated (25 mM) glucose concentration for various periods of time (2, 4, 24 and 48 h). Superoxide was then visualized under a confocal fluorescent microscope (Zeiss LSM 510) using appropriate filters.

### RT-PCR analysis

Eight hundred ng of total RNA in a 20 µl reaction were used for cDNA synthesis using oligo (dT)18 according to the manufacturer's procedure (First strand cDNA synthesis kit for RT-PCR, Roche Applied Science, Rotkreuz, Switzerland). The equivalent of 2 to 20 ng original total RNA was used for quantitative PCR amplification using the 2 x brilliant SYBR Green QPCR Master Mix (Stratagene) and 0.5 µM forward and reverse primer pair, designed to span an intron of the target gene. We used Quantitec primer assay (Qiagen, Switzerland) for Nox2 qPCR analysis. Real-time PCR was performed in multiple replicate in the Mx3000PTM system (Stratagene) with the conditions described in Table S1.

### GSH and ATP analysis

Twenty µg of total protein lysates, isolated from the retina of sham operated (Ctl), hypoglycemic (Hypo) and euglycemic (Eugly) mice, sacrificed 48 h post-clamp, were used for the measurement of GSH retinal content. 661W cells were cultured as described above, and GSH content was measured with Bioxytech GSH/GSSG-412 kit (#21040; OxisResearch, Beverly Hills, CA, USA) accordingly to the protocol. ATP content was measured with a luminescence assay kit (ATPLite, PerkinElmer-BioSignal, Montreal, QC, Canada) as described by the manufacturer. Briefly, cells were cultured in 96-well plate at various glucose concentrations for 24 h or at 2 mM glucose for different periods of time, then lysis buffer containing substrate was added directly to the cell and luminescence was measured on an EnVision multilabel reader (PerkinElmer- BioSignal, Montreal, QC, Canada), after 30 minutes of incubation.

### Statistical Analysis

All results were expressed as means ± SEM of the indicated number of experiments. Statistical significance was calculated with the Student's *t* test.

## Results

### Acute 5 hours hypoglycemia induced retinal cell death in mouse

**Figure 1:**
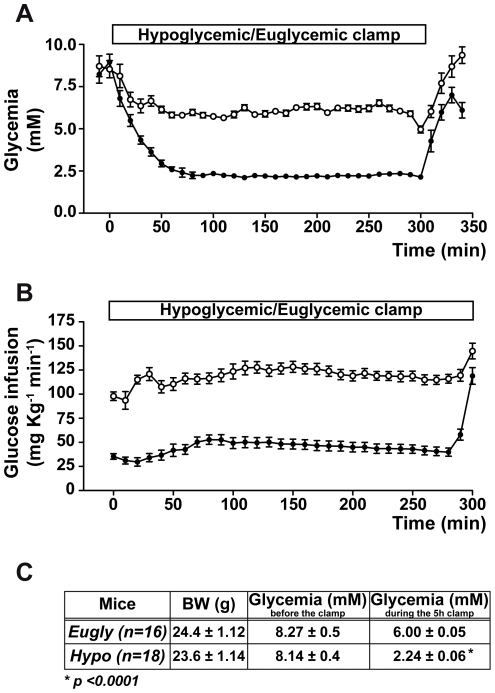
Insulin-induced hypoglycemia in C57BL/6 mouse. **A**) Graphic representation of plasma glucose levels; **B**) glucose infusion rates during the hyperinsulinemic/hypoglycemic clamp (black circle) and the control hyperinsulinemic/euglycemic clamp (white circle). **C**) Mouse characteristics before and during the clamp.

**Figure 2:**
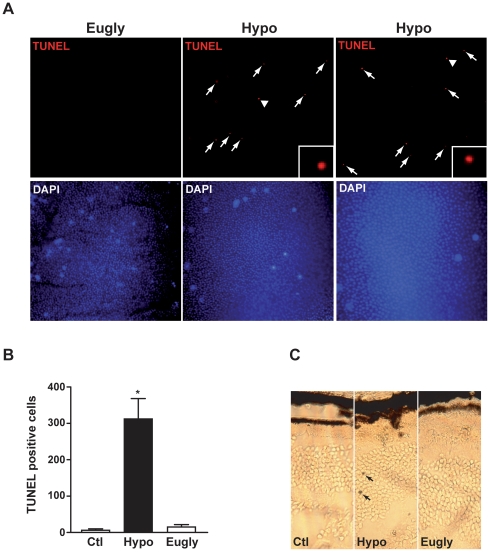
Acute hypoglycemia induced cell death in mouse retina. **A**) Flat-mounted retinas were isolated 48 h after the clamp, stained for cell death by TUNEL assay and DAPI counter coloration was performed. White arrows show TUNEL positive cells in hypoglycemic condition. **B**) Quantification of TUNEL positive cells was performed under a fluorescence microscope on retinal flat-mounts. Results are expressed as mean ± SEM of 3 different retinas for each group, * p<0.006 Hypo *vs.* Eugly. **C**) Ten µm-embedded frozen sections of enucleated eyes isolated from control (Ctl), hypoglycemic (Hypo) and euglycemic (Eugly) animals were stained for cell death by colorimetric TUNEL system. Using this procedure, apoptotic nuclei are stained dark brown (black arrow). A representative region of three different isolated retinas is shown.

To analyze the effect of well-controled hypoglycemia on mouse retina, we performed a hyperinsulinemic/hypoglycemic clamp (Hypo) to induce a five-hour hypoglycemia in mouse. We used sham-operated mice (Ctl) and mice treated with a hyperinsulinemic/euglycemic clamp (Eugly) as control groups. The latter group of mice received comparable amounts of insulin but was injected with glucose in order to maintain normal glycemia. Comparison of these two groups allowed us to analyze the specific effect of hypoglycemia. We monitored glycemia during the whole clamp (Fig. 1A). Glucose infusion, necessary to maintain either the hypoglycemia at 2.2 mM or the euglycemia at 6 mM, is shown in figure 1B. Body weight and glycemia before the clamp were similar for both groups (Fig. 1C), while a significant expected difference in glycemia was observed during the clamp (6.00±0.05 and 2.24±0.06 for euglycemic and hypoglycemic mice, respectively). We first isolated flat-mount retinas 48 h after the clamp, and performed a TUNEL staining in each group. Figure 2A shows TUNEL-positive cells in the retina from hypoglycemic mice. Very few positive cells were observed in euglycemic (Fig. 2A) and control sham-operated mice (data not shown). We quantified apoptotic cells from three flat-mount retinas isolated from each group and obtained 312±56 TUNEL-positive cells after acute hypoglycemia (Fig. 2B) while few positive cells (15±6 for Eugly and 6±4 for Ctl) were detected in the two control groups. The majority of TUNEL positive cells were localized in the outer nuclear layer (ONL) and ganglion cell layers (GCL), as observed by confocal microscopy. To more precisely define which compartment of the retina was affected, we performed TUNEL assay on 10-µm-embedded frozen sections. Figure 2C shows two TUNEL positive cells in the ONL of hypoglycemic retina, but we also observed TUNEL positive cells in the GCL (data not shown).

### Low glucose culture conditions induced 661W photoreceptor cell death

**Figure 3:**
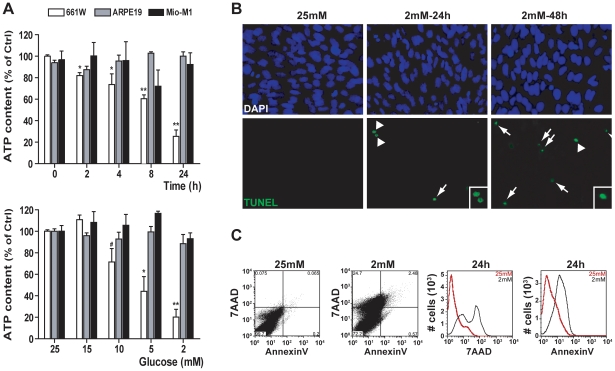
Low glucose concentration induced 661W cell death. 661W, ARPE19 and Mio-M1 cells were cultured as mentioned in material & methods, then **A**) cells were incubated at 2 mM glucose for different time periods or for 24 h at different glucose concentrations. ATP content was measured and found decreased in 661W cells (white box) after exposure to low glucose concentrations while no change was observed for ARPE19 (gray box) and Mio-M1 cells (black box). Results are expressed as mean ± SEM of 3 experiments, # p<0.05, * p<0.002 and ** p<0.0001 *vs.* control (25 mM glucose or time 0). **B**) 661W cells were cultured as mentioned above, then cell death was analyzed by TUNEL assay, performed 24 or 48 h after low glucose exposure followed by DAPI counter coloration. White arrows indicate TUNEL positive cells and condensed nuclei. TUNEL analysis was representative of 3 distinct experiments. **C**) 661W cells were stained with AnnexinV-FITC and 7-AAD and analyzed by FACS after exposure to low glucose for 24 h.

To better characterize the retinal cell death induced by low glucose, we cultured different types of retinal cells (transformed photoreceptor cells: 661W; retinal pigment epithelial cells: ARPE19 and Müller cells: Mio-M1) at low glucose conditions (2 mM) for various periods of time or for 24 h at diverse glucose concentrations (Fig. 3A). While the ATP content did not change at low glucose concentrations in ARPE19 and Mio-M1 cells (Fig. 3A), we observed a dose- and time-dependent decrease of the ATP content in the 661W cell line cultured at low glucose. TUNEL assay performed on 661W cells showed a time dependant effect of low glucose on cell death (Fig. 3B).

We then cultured these cells at low glucose concentration for 24 h and measured cell death by fluorescein isothiocyanate (FITC)-conjugated AnnexinV and 7-Aminoactinomycin D (7-AAD) labeling. Cells undergoing apoptosis exhibited disorganization of the plasma membrane, followed by the externalization of certain phospholipids such as phosphatidyl serine. AnnexinV-positive and 7-AAD-negative cells showed early stage of apoptosis, whereas cells positive for both 7-AAD and AnnexinV showed necrotic or late apoptosis due to compromised plasma membrane permeability (Fig. 3C). We could also observe late apoptosis occurring after 24 h in cells exposed to low glucose concentration.

### Low glucose-induced retinal cell death was a Caspase 3 dependent process

**Figure 4:**
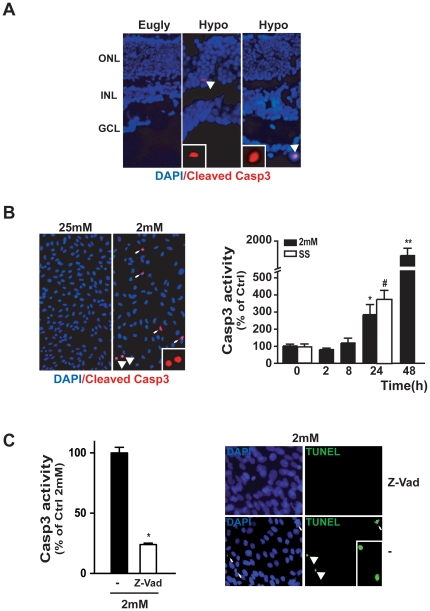
Caspase 3 was activated in the retina of hypoglycemic mice and in 661W cells incubated at low glucose condition. **A**) Immunohistological staining with cleaved Caspase 3 antibody showed positive cells (white arrow) in the outer nuclear (ONL) and ganglion cell layers (GCL) of the hypoglycemic mice retina. Counterstaining with DAPI was performed to identify the retinal cell layers. Results were representative of three observed retinas for each group. **B**) Similar immunostaining of cleaved Caspase 3 (white arrows) was performed on 661W cells, cultured during 48 h at 2 mM glucose and additional Caspase 3 activity was assessed in a time course at low glucose (2 mM) experiment, using a 24 h serum starved (SS) cultured condition as positive control. Results are expressed as mean ± SEM of 3 experiments, *p<0.006 and **p<0.0001 *vs.* Ctrl (time 0). C) Measures of Caspase 3 activity in 661W cells cultured for 48 h at 2 mM glucose in absence or presence of Z-VAD-FMK inhibitor (10 µM). Results are expressed as mean ± SEM of 3 experiments, *p<0.0001. TUNEL assay was performed on similar conditions; white arrows indicated TUNEL positive cells.

As apoptosis is often associated with caspase 3 activity, we tested the activation of this enzyme in the retina of hypoglycemic and euglycemic mice. We isolated whole eyes and fixed them prior to analysis for cleaved caspase 3. Figure 4A shows positive cells in the ONL and GCL of hypoglycemic mice, while no positive cells for cleaved caspase 3 were observed in the retina of euglycemic mice. Similar results were observed *in vitro* when photoreceptor 661W cells were cultured for 24 h at low glucose (2 mM) concentration (Fig. 4B). Caspase 3 activity induced by low glucose was time-dependent as shown by the large increase of caspase 3 activity after 48h, as measured by luminescent assay. We were able to observe the induction of caspase 3 activity when the cells were serum starved (SS) during 24 h as already described by Mackey *et al.* [19]. We then decided to confirm the role of Caspase 3 in low glucose-induced 661W cell death, by testing the effect of an inhibitor. Figure 4C showed that Z-VAD-FMK decreased Caspase 3 activity by 70% and blocked 661W apoptosis, as assessed by TUNEL analysis.

### Low glucose induced mitochondrial superoxide production

**Figure 5:**
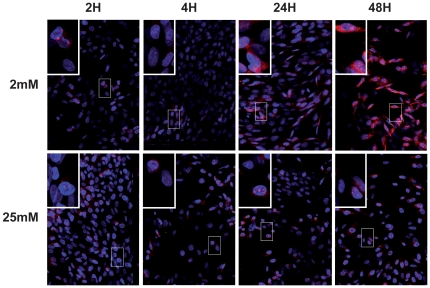
Mitochondrial superoxide production was detected after exposure of 661W cells to low glucose. Detection of low glucose-induced peroxide production was performed on 661W photoreceptor cells with the MitoSOX™ dye. 661W cells were cultured at 25 mM and 2mM glucose for diverse periods of time. Fluorescence intensity was visualized under a confocal microscope with appropriate filters.

We then tested for low glucose induction of reactive species (RS) as these compounds are regularly associated with programmed cell death. We used the selective MitoSOX™ red indicator to detect superoxide production. Exposure of 661W photoreceptor cells to low glucose concentration induced mitochondrial superoxide production after 24 h that increased after 48 h (Fig. 5). Some nuclei localization of fluorescent dye was also seen. This is most likely due to the use of high concentration.

### Reduced glutathione (GSH) content played key role in cell death

**Figure 6:**
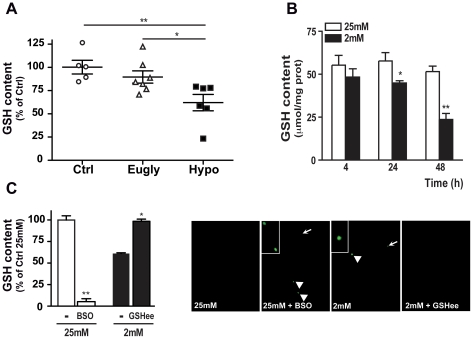
GSH content was decreased in low glucose conditions. **A**) GSH content was measured in protein lysates obtained from retina of control (white circle), euglycemic (white triangle) and hypoglycemic (black square) mice. Results were expressed as percent of control and as mean ± SEM of 5 to 7 samples. *p<0.025 vs. Eugly and **p<0.01 vs. Ctrl. **B**) 661W cells were cultured at low (2 mM) and high (25 mM) glucose conditions and GSH content was measured at diverse periods of time. Results were expressed as mean ± SEM of 3–5 experiments, *p<0.03 and **p<0.0002 vs. 25 mM glucose. **C**) GSH content was measured in 661W cells cultured for 48 h at 25 mM or 2 mM glucose, in absence or in presence of 200 µM buthionine sulphoximine (BSO) or 1mM extracellular glutathione ethyl ester (GSHee). At the same time we measured cell death by TUNEL assay in each condition. White arrows indicated TUNEL positive cells. Results are expressed as mean ± SEM of 3 experiments; *p<0.003 vs. 2 mM glucose without GSHee.

Several recent studies implicated the GSH scavenger protein in apoptotic processes [1], [20], [21]. To analyze in detail the key role of GSH content in the process of cell death, we measured its concentration in the retina of the three mouse groups. GSH content was significantly decreased in hypoglycemic animals in comparison with sham operated and euglycemic mice (Fig. 6A). A similar result was observed in 661W cells with a 50% decrease of GSH concentration 48 h after low glucose exposure (Fig. 6B). Depletion of GSH content by inhibition of glutathione synthase with buthionine sulphoximine (BSO) compound induced apoptosis at 25 mM glucose, while restoration of GSH concentration to “normal” levels by adding external GSH (glutathione ethyl ester; GSHee), at low glucose condition was accompanied by a blockage of cell death (Fig. 6C).

**Figure 7:**
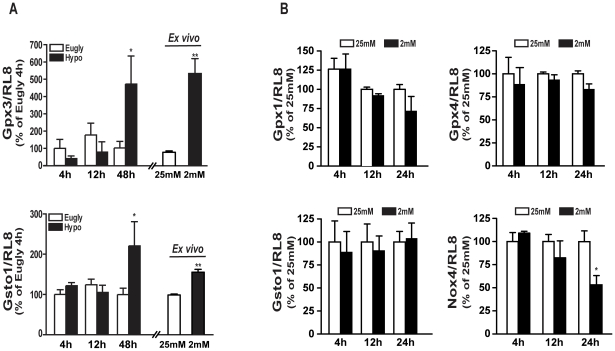
Expression of enzymes implicated in GSH homeostasis was modulated by glucose concentration. **A**) We tested the expression of two genes, Gpx3 and Gsto1, in the retina of hypoglycemic and euglycemic animals 48 h after the clamp. We were able to show, by RT-qPCR, the induction of both genes in hypoglycemic conditions. In addition, we incubated retinal explants isolated from C57bl/6 mice for 48 h at low (2 mM) and high (25 mM) glucose conditions and measured Gpx3 and Gsto1 expression in the whole retina. *RL8* (ribosomal protein L8) was used as internal control to normalize RNA expression and results are expressed as mean ± SEM of 3 (4 and 12 h) to 8 (48 h) retinas, *p<0.03 and as mean ± SEM of 4 to 6 isolated retina **p<0.02 vs. 25 mM glucose. **B**) We tested by RT-qPCR the expression of Gpx1, Gpx4, Gsto1 and Nox4 in 661W cells after incubation for diverse periods of time (4, 12 and 48 h) at low (2 mM) and high (25 mM) glucose conditions. *RL8* was used as internal control to normalize RNA expression and results are expressed as mean ± SEM of 3 experiments in triplicate.

To decipher which genes were involved in the low glucose-induced cell death and GSH decrease, we tested the mRNA expression of two enzymes playing a role in the GSH homeostasis: the glutathione peroxidase 3 (Gpx3) functioning in the detoxification of hydrogen peroxide and the glutathione S-transferase omega 1 (Gsto1) serving a protective role by adding GSH to proteins exported from the cell. We observed an increased expression for both genes at 48 h by q-PCR analysis (5 fold for Gpx3 and 2.2 fold for Gsto1) while no change in mRNA expression was visible 4- and 12-h after the clamp (Fig. 7A). Analysis of retinal explants showed that *in vitro* exposure to low glucose (2 mM) for a 48-h period significantly increased the expression of Gpx3 and Gsto1 to the same extent as *in vivo* hypoglycemia (Fig. 7A). We then tested for the expression of members of the peroxidase family in 661W cells cultured at low glucose concentrations. While Gpx3 was not expressed in those cells, expression of Gpx1 and Gpx4, two other members of the Gpx family, did not change. Neither was Gsto1 expression affected by low glucose condition in 661W cells (Fig. 7B). However, we observed a down-regulation of the expression of NADPH oxidase 4 (Nox 4), an enzyme involved in the control of NADPH level and GSH homeostasis in the cell. We also observed a slight, but not significant, increase of NADPH oxidase 2 (Nox2) in both, the *in vivo* and *in vitro* situations of low glucose (Figure S2).

## Discussion

Glucose is essential for retinal function and glycemic excursions may have harmful effects on vision. In diabetic patients, deleterious effects of hyperglycemia have been well documented and chronic hypoglycemia has been shown to affected retinal cells in an animal model [9] and may induce cone cell death in retinitis pigmentosa [10]. In general, type I diabetic patients suffer at least two episodes of hypoglycemia per week, and one or more severe hypoglycemia, often with seizure or coma, per year [3].

In this study, we concentrated our investigation on acute hypoglycemia, which is equivalent to severe hypoglycemia occurring in patients, knowing that cerebral and retinal glucose concentration approaches 0 mM when blood glucose concentration falls below 2 mM [22]. We showed that acute insulin-induced hypoglycemia in mouse (blood glucose at 2.2 mM) led to retinal apoptosis through the activation of caspase 3 and the modulation of retinal GSH content. Despite positive cells for cleaved caspase3 were detected in retinas of hypoglycemic animals, further experiments with caspase inhibitor will be necessary to clearly implicate this enzyme in the apoptotic process observed *in vivo*. *In vitro* experiments performed on 661W photoreceptor cells confirmed both the activation of caspase 3 and the decrease of GSH; additionally we were able to show mitochondrial superoxide production.

Increased production of mitochondrial reactive species (RS) by hyperglycemia is recognized as a major cause of the clinical complications associated with diabetes and obesity. This production is otherwise linked to a down-regulation of the GSH pool, via activation of aldolase [1], and to the activation of caspase 3, leading to neuronal dysfunction through N-methyl-D-aspartate (NMDA) receptors [23]. It is interesting to notice that we observed, in addition to GSH down regulation and caspase 3 activation, a production of mitochondrial superoxide. These results are supported by several studies showing an increase of superoxide production on hippocampal neurons cultured under hypoglycemic conditions [24] and in neurons after glucose deprivation [25]. Moreover, *in vivo* studies recently showed production of mitochondrial RS in newborn brain during acute hypoglycemia [26] and in insulin-induced hypoglycemic stress in healthy subject [27].

Our results suggest that GSH level is a key player in retinal apoptosis at least in 661W cells, as a decrease of GSH either by low glucose or by BSO treatment correlated with cell death; while replenishment of GSH blocked the apoptotic process. Further *in vivo* experiments will be needed to clearly demonstrate the implication of GSH decrease in cell death in hypoglycemic conditions. The low glucose-induced GSH decrease is supported by the work of Winkler *et al.* who observed a similar decrease when they studied the effect of diamide oxidant on isolated rat retina [28]. Interestingly, a decrease of GSH level was also observed in the retina of streptozotocin-induced diabetic mice [29] and rats [30], and restored by an over-expression of the antioxydant mitochondrial superoxide dismutase (MnSOD) protein [29].

GSH is the predominant cellular non-protein thiol and is usually up-regulated as an adaptative response to oxidative stress in order to maintain a stable redox status. The key role of this tripeptide may be more relevant in the retina, because of the high oxidative metabolic activity occurring in this tissue. In addition, GSH is involved in many cellular functions including apoptosis [11], [12]. On the one hand, it has been shown that GSH depletion due to buthionine sulphoximine (BSO)-induced inhibition of GSH synthesis, sensitizes cells to hyperoxia and H_2_O_2_ [21] or leads to genome rearrangement [31]. On the other hand, addition of extracellular glutathione ethyl ester (GSHee) to restore normal GSH level, or the use of non-toxic analogue of cysteine, N-acetyl cysteine (NAC), to increase GSH synthesis, reduces or counteracts oxidative damage [31] and prevents retinal photooxidative damage induced by intense light [32].

How is the GSH cell content decreased in low glucose conditions? In mouse, hypoglycemia induced up-regulation of Gpx3 and Gsto1, which may partially explain the 40% decrease of retinal GSH scavenger seen in our experiments. We observed a large increase of Gpx3 mRNA expression in the retina of hypoglycemic animals and our results are in accordance with a recent study published by Miranda *et al.* showing an inhibitory effect of glucose on Gpx activity, *in vitro* but also in alloxan-induced diabetic animals [33]. Further promoter analyses will be necessary to confirm and define more precisely the sequence responsible for the transcriptional effect of glucose on Gpx3 promoter. We also observed an increase of Gsto1 expression in hypoglycemic mice, but no clear glucose-induced modulation of glutathione transferase has been described in the literature. A recent study showed that Gsto1 overexpression protects Hela cells against cisplatin-induced apoptosis [34]. Yet, this enzyme is able to modify protein function via s-glutathionylation, a process critical for preventing irreversible oxidation of proteins [35] and therefore might prevent low glucose-induced apoptosis. The use of thiol groups either by this latter mechanism of glutathionylation or by peroxidation of RS leads to a decrease of GSH pool. Normally, both reactions are necessary to counteract oxidative stress induced by low glucose in the retina. Further experiments by analyzing 8-hydroxyguanosine (8-OHdG) production, resulting from oxidative DNA damage, directly in the retina of hypoglycemic mouse, will be necessary to challenge low glucose induction of superoxide *in vivo*. Furthermore, the decrease of GSH content could be the result of a down-regulation of GSH synthesis. These ATP-dependent reactions could be altered in hypoglycemic (low ATP) conditions and/or changed because of a decrease in the glutamate or cysteine cell content, which are modulated by glucose [35]. Moreover, glutamate uptake is an active ATP-dependent process and the rate of glutamate refilling into the vesicles is reduced in the absence of glucose [36].

The decrease of cellular GSH content observed in the 661W cells is more difficult to explain because it was not dependent on Gpx3, which we found not to be expressed in these cells. We tested other members of the peroxidases family, i.e. Gpx1 and Gpx4 that were described to protect retina against oxidative damage [20], but could not observe any modification of their expression. Gsto1 was most likely not involved, as its expression was not modified by low glucose exposure. The decrease of Keap1 and Nrf2 (Figure S1C) may possibly explain GSH depletion, because many genes involved in GSH synthesis (eg. glutamate cysteine ligase (GCL), glutathione synthase (GS) and glutathione reductase (GR)) are regulated by Nrf2 nuclear factor ([37], [38]). On the other hand, we did not observed any modification of expression of these two genes in hypoglycemic animals (Figure S1A/S1B). Further analysis at protein level will be necessary to detail the role of both transcription factors in our hypoglycemic model.

Oxidation of NADPH to NADP^+^ by producing H_2_O_2_ could lead to an important reduction of GSSG back to GSH in presence of GR. We observed a decrease of Nox4 expression and a slight, but not significant, increase of Nox2 (Figure S2) in low glucose condition. These results are not in accordance with previous published studies showing an increase of neuronal NADPH oxidase activity due to glucose reperfusion during a very-short term hypoglycemia [39]. Both enzymes have been described to contribute to 661W cell survival and a modification in their expression could sensitize cells to apoptosis [40]. Change in expression of NADPH oxidases would probably not clarify the GSH reduction, but would explain cell death. However, absence or reduction of glucose may lead to a decrease in multiple-NADPH production pathways which might partially explain a reduction of GSH [28]. It would be interesting to test if low glucose could inhibit the pentose shuttle, decrease NADPH level and thus, control GSH cell content. Further analysis will be needed to decipher the specific role of NADPH oxidases (Nox 2 and Nox4) in the mechanisms induced by low glucose in the 661W photoreceptor cell line and in hypoglycemic animals.

We also tested protein expression of hypoxia-inducible factor (HIF) 3 α, because it has been described as key regulator in glucose metabolism [41]. Heidbreder *et al.* recently described an *in vivo* induction of this gene by insulin-induced hypoglycemia and glucoprivation. We did not observe any HIF3 α increase in our hypoglycemic mouse model (Figure S3A), but control groups were different. We used a hyperinsulinemic/euglycemic clamp, while Heidbreder *et al.* used saline injected animals as control in comparison to hyperinsulinemic/hypoglycemic clamp and insulin injected, respectively. This may explain the difference with our results and justify further analysis. However, in low glucose (2 mM) cultured 661W cells we observed an increase of HIF3 α (Figure S3B), which is similar to Heidbreder's observation in HT-22 cells cultured at 5 mM [41]. We clearly need further studies to implicate this protein in GSH depletion, ROS production and caspase 3-induced apoptosis.

Our hypothesis is that sensitivity of retinal cells to stress (in our case low glucose) is directly dependent on the GSH level. Enzymes involved in GSH homeostasis try to counteract apoptosis by adaptation to harmful environmental conditions. This leads to a strong decrease of GSH that is deleterious for retinal cells. Modulation of GSH/GSSG ratio is crucial to maintain normal redox state and cell function. An increase of GSH will protect cells against oxidative stress, while a low glucose-induced depletion of GSH will induce an apoptotic process.

The key role of GSH in the retina is highlighted by the retinal dystrophy observed in 2 sisters with GS deficiency [42]. In those patients, the level of GSH content was 80% lower than in normal subjects, and about 25% of patients affected by GSH deficiency developed retinitis pigmentosa, retinal dystrophy, lens opacities or decrease of visual acuity [43].

Many of the effects observed in our hypoglycemic conditions were similar to those observed in hyperglycemic conditions [1]. These results strongly suggest that glycemic excursions may not be harmless. A strict control of glycemia, to fence not only hyperglycemia but also hypoglycemia, is critical for diabetic patients for the maintenance of a good retinal function and vision. Treatment of diabetic patients with antioxidant compounds may, in addition, help to maintain stable redox within the cells and reduce retinal cell death induced by hypoglycemia.

## Supporting Information

Figure S1 ***Nrf2*** **and** ***Keap1*** **expression after exposure to low glucose.** **A**) We tested the expression of *Nrf2* and *Keap1* in the retina of hypoglycemic and euglycemic animals 48 h after the clamp. We were not able to show any modification of gene expression in hypoglycemic condition. Results are expressed as % of control (Eugly) ± SEM of 5 retinas. **B**) In addition, we obtained similar results when we incubated retinal explants isolated from C57bl/6 mice for 48 h at low (2 mM) and high (25 mM) glucose conditions. *RL8* was used as internal control to normalize RNA expression and results are expressed as % of control (25 mM) ± SEM of 8 retinas. **C**) We tested the expression of *Nrf2* and *Keap1* in 661W cells after incubation for diverse periods of time (8, 24 and 48 h) at low (2 mM) and high (25 mM) glucose conditions. *RL8* was used as internal control to normalize RNA expression and results are expressed as % of control (25 mM) ± SEM of 2 experiments in triplicate, *p<0.0001.
(TIF)

Figure S2 **Expression of** ***Nox2*** **after exposure to low glucose.** **A**) We tested the expression of *Nox2* in the retina of hypoglycemic and euglycemic animals 48 h after the clamp. We observed a small, not significant increase of gene expression in hypoglycemic condition. *RL8* was used as internal control to normalize RNA expression and results are expressed as % of control (Eugly) ± SEM of 4 retinas. **B**) In addition, we obtained similar results when we tested *Nox2* expression in 661W cells cultured at low (2 mM) and high (25 mM) glucose conditions for various periods of time. RL8 was used as internal control to normalize RNA expression and results are expressed as % of control (25 mM) ± SEM of 3 retinas.
(TIF)

Figure S3 **HIF3 α protein expression in hypoglycemic conditions.** **A**) We tested the expression of HIF3 α in the retina of hypoglycemic and euglycemic animals 48 h after the clamp and found no variation in hypoglycemic condition. Tubulin was used as internal control to normalize protein expression and results are expressed as % of control (Eugly) ± SEM of 4 retinas. **B**) We observed an increase of HIF3 α in photoreceptor 661W cells cultured at low (2 mM) glucose condition for 48 hours. Tubulin was used as internal control to normalize protein expression and results are expressed as % of control (25 mM) ± SEM of 5 retinas. *p<0.002.
(TIF)

Table S1 **qPCR conditions with primers.**
(TIF)
