# Supplementary figures and images for: Acute Hypoglycemia Induces Retinal Cell Death in Mouse
Source: PLoS One. 2011 Jun 27;6(6):e21586. doi: 10.1371/journal.pone.0021586 (PMC3124528; doi:10.1371/journal.pone.0021586)

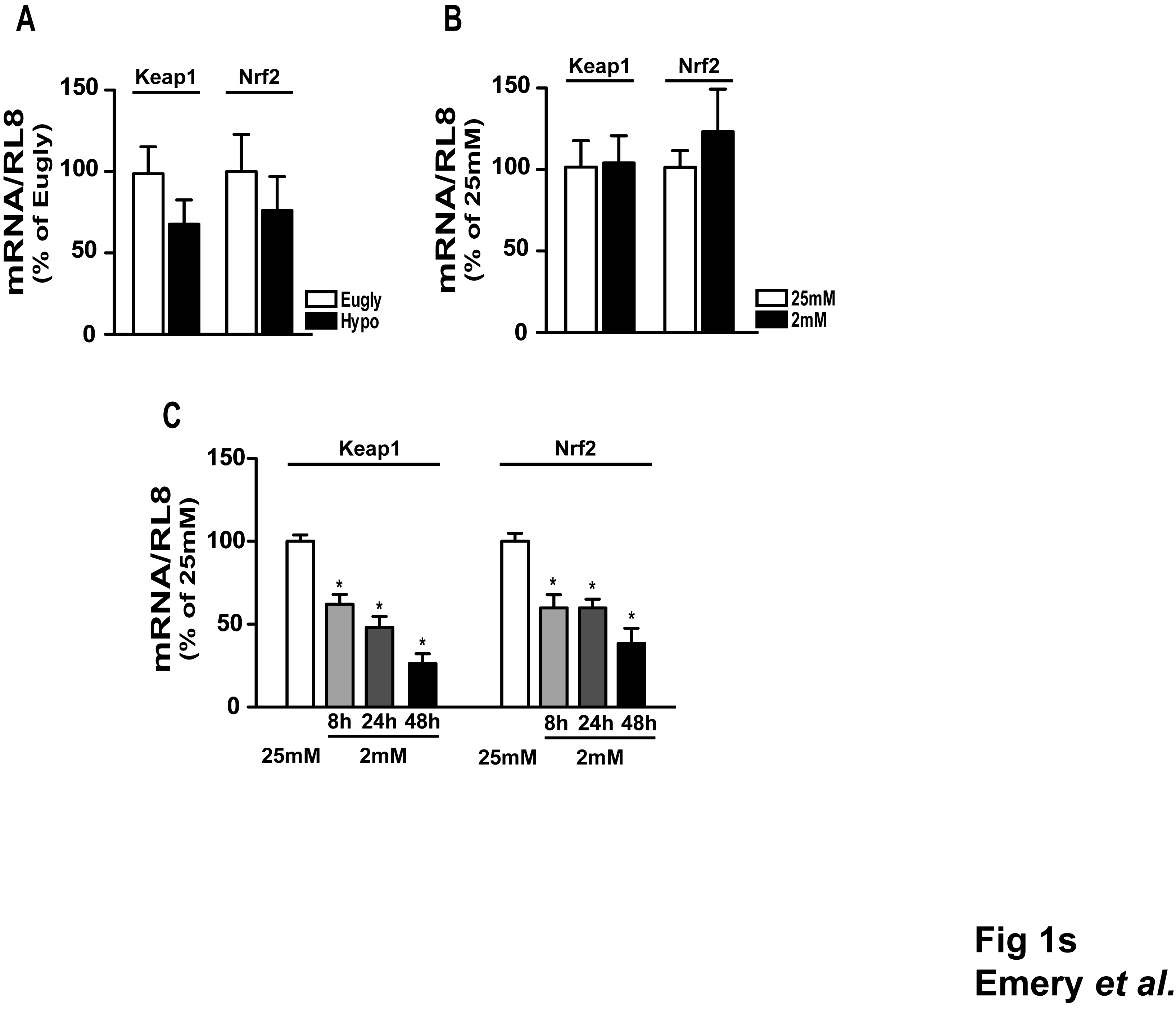

Supplement: Figure S1 — Nrf2 and Keap1 expression after exposure to low glucose. A) We tested the expression of Nrf2 and Keap1 in the retina of hypoglycemic and euglycemic animals 48 h after the clamp. We were not able to show any modification of gene expression in hypoglycemic condition. Results are expressed as % of control (Eugly) ± SEM of 5 retinas. B) In addition, we obtained similar results when we incubated retinal explants isolated from C57bl/6 mice for 48 h at low (2 mM) and high (25 mM) glucose conditions. RL8 was used as internal control to normalize RNA expression and results are expressed as % of control (25 mM) ± SEM of 8 retinas. C) We tested the expression of Nrf2 and Keap1 in 661W cells after incubation for diverse periods of time (8, 24 and 48 h) at low (2 mM) and high (25 mM) glucose conditions. RL8 was used as internal control to normalize RNA expression and results are expressed as % of control (25 mM) ± SEM of 2 experiments in triplicate, *p<0.0001. (TIF) [file pone.0021586.s001.tif]

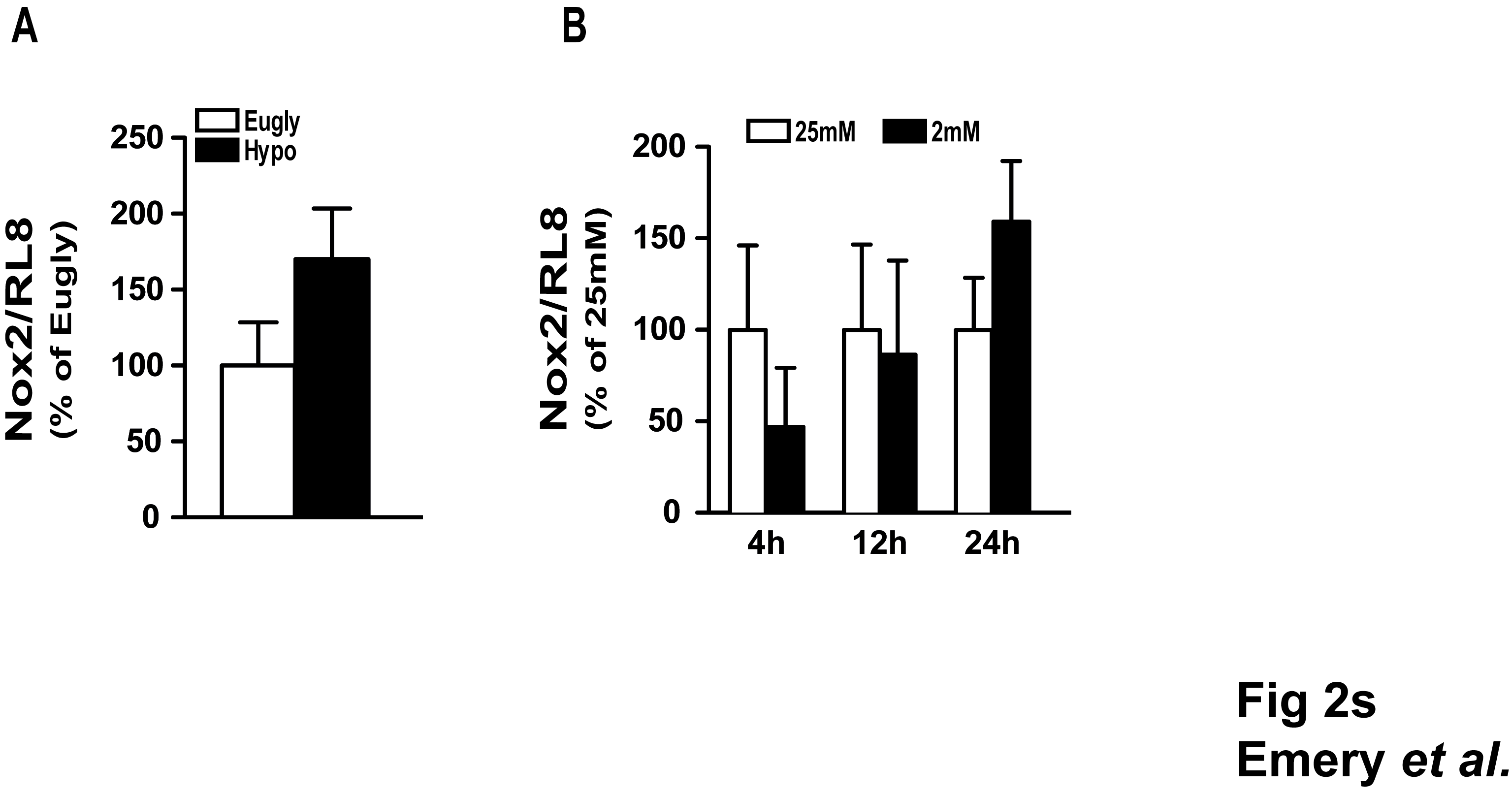

Supplement: Figure S2 — Expression of Nox2 after exposure to low glucose. A) We tested the expression of Nox2 in the retina of hypoglycemic and euglycemic animals 48 h after the clamp. We observed a small, not significant increase of gene expression in hypoglycemic condition. RL8 was used as internal control to normalize RNA expression and results are expressed as % of control (Eugly) ± SEM of 4 retinas. B) In addition, we obtained similar results when we tested Nox2 expression in 661W cells cultured at low (2 mM) and high (25 mM) glucose conditions for various periods of time. RL8 was used as internal control to normalize RNA expression and results are expressed as % of control (25 mM) ± SEM of 3 retinas. (TIF) [file pone.0021586.s002.tif]

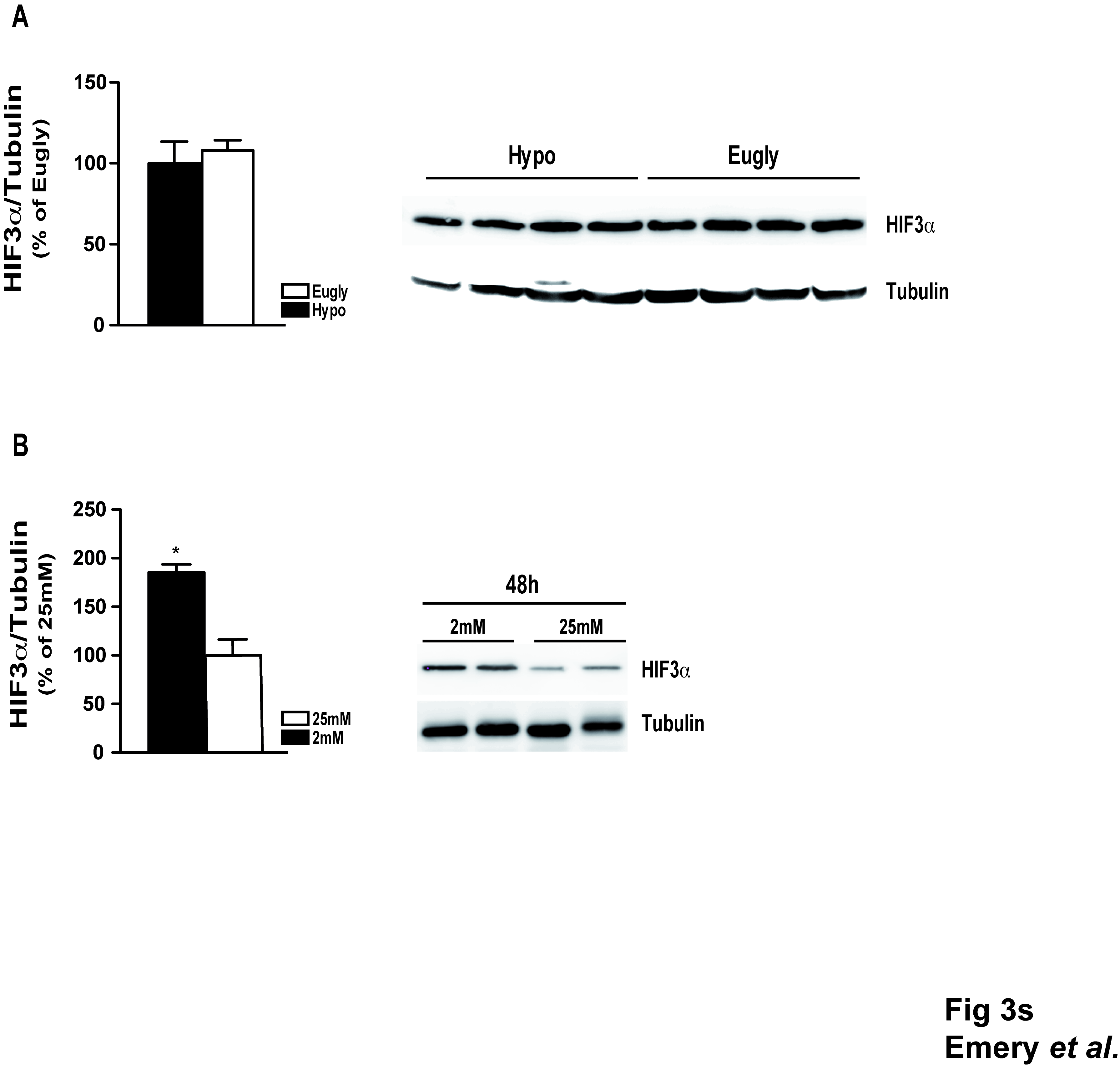

Supplement: Figure S3 — HIF3 α protein expression in hypoglycemic conditions. A) We tested the expression of HIF3 α in the retina of hypoglycemic and euglycemic animals 48 h after the clamp and found no variation in hypoglycemic condition. Tubulin was used as internal control to normalize protein expression and results are expressed as % of control (Eugly) ± SEM of 4 retinas. B) We observed an increase of HIF3 α in photoreceptor 661W cells cultured at low (2 mM) glucose condition for 48 hours. Tubulin was used as internal control to normalize protein expression and results are expressed as % of control (25 mM) ± SEM of 5 retinas. *p<0.002. (TIF) [file pone.0021586.s003.tif]

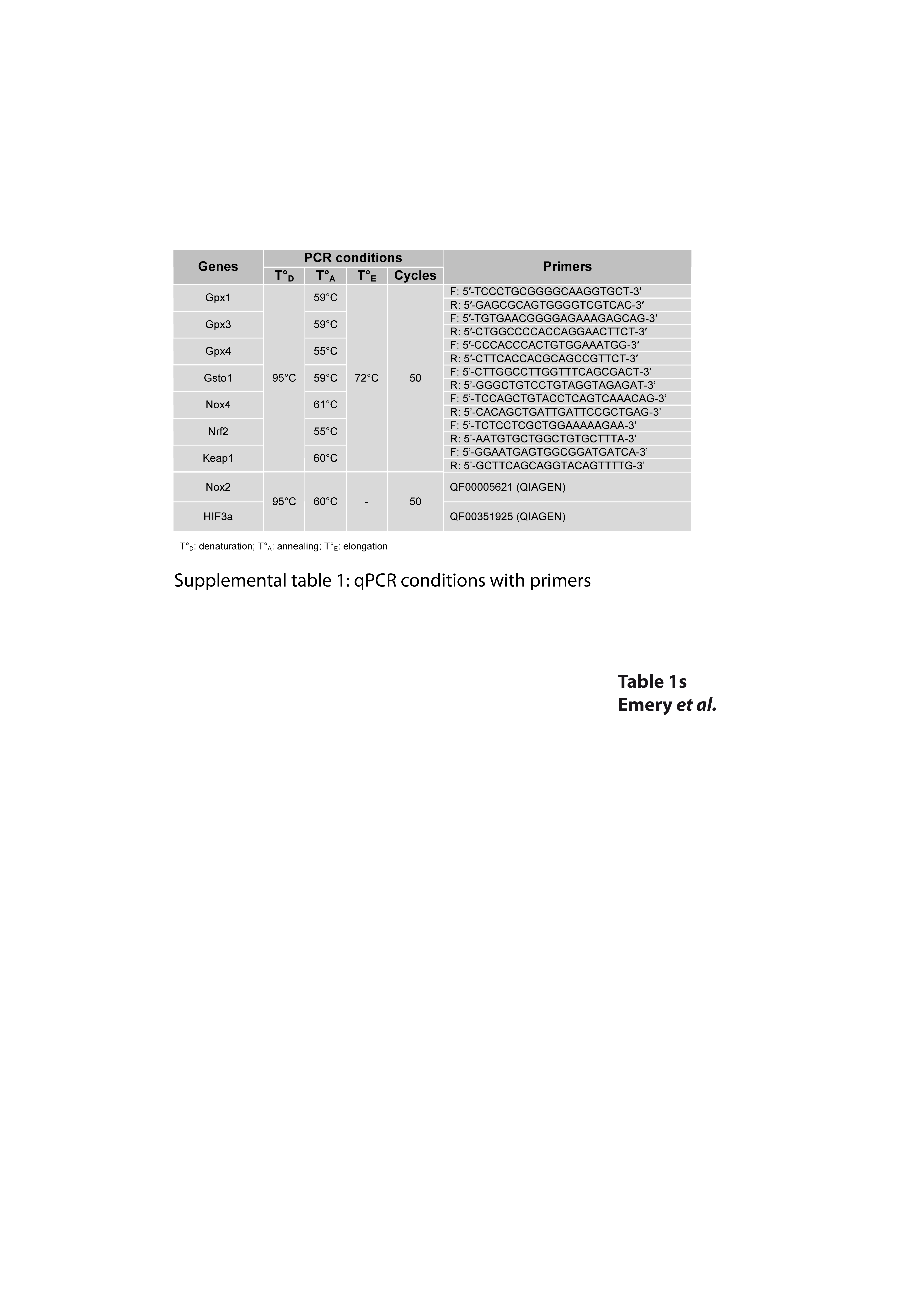

Supplement: Table S1 — qPCR conditions with primers. (TIF) [file pone.0021586.s004.tif]
